# Combination Treatment With Remdesivir and Ivermectin Exerts Highly Synergistic and Potent Antiviral Activity Against Murine Coronavirus Infection

**DOI:** 10.3389/fcimb.2021.700502

**Published:** 2021-07-30

**Authors:** Yu Ling Tan, Kevin S. W. Tan, Justin Jang Hann Chu, Vincent T. Chow

**Affiliations:** ^1^Infectious Diseases Translational Research Program, Department of Microbiology and Immunology, National University of Singapore, Singapore, Singapore; ^2^Healthy Longevity Translational Research Program, Department of Microbiology and Immunology, National University of Singapore, Singapore, Singapore

**Keywords:** coronavirus, murine hepatitis virus, remdesivir, chloroquine, ivermectin, doxycycline, combination treatment, RAW264.7 macrophage cells

## Abstract

The recent COVID-19 pandemic has highlighted the urgency to develop effective antiviral therapies against the disease. Murine hepatitis virus (MHV) is a coronavirus that infects mice and shares some sequence identity to SARS-CoV-2. Both viruses belong to the *Betacoronavirus *genus, and MHV thus serves as a useful and safe surrogate model for SARS-CoV-2 infections. Clinical trials have indicated that remdesivir is a potentially promising antiviral drug against COVID-19. Using an *in vitro* model of MHV infection of RAW264.7 macrophages, the safety and efficacy of monotherapy of remdesivir, chloroquine, ivermectin, and doxycycline were investigated. Of the four drugs tested, remdesivir monotherapy exerted the strongest inhibition of live virus and viral RNA replication of about 2-log_10_ and 1-log_10_, respectively (at 6 µM). Ivermectin treatment showed the highest selectivity index. Combination drug therapy was also evaluated using remdesivir (6 µM) together with chloroquine (15 µM), ivermectin (2 µM) or doxycycline (15 µM) – above their IC50 values and at high macrophage cell viability of over 95%. The combination of remdesivir and ivermectin exhibited highly potent synergism by achieving significant reductions of about 7-log_10_ of live virus and 2.5-log_10_ of viral RNA in infected macrophages. This combination also resulted in the lowest cytokine levels of IL-6, TNF-α, and leukemia inhibitory factor. The next best synergistic combination was remdesivir with doxycycline, which decreased levels of live virus by ~3-log_10_ and viral RNA by ~1.5-log_10_. These results warrant further studies to explore the mechanisms of action of the combination therapy, as well as future *in vivo* experiments and clinical trials for the treatment of SARS-CoV-2 infection.

## Introduction

SARS-CoV-2 first emerged in China in December 2019, causing numerous cases of pneumonia, and it was subsequently confirmed to be a novel coronavirus in January 2020. Since then, this highly infectious virus together with its emerging variants have transcended international borders and spread rapidly across the globe, resulting in high infection rates and millions of deaths worldwide. Coronaviruses are positive-sense, single-stranded RNA viruses with a distinct crown-like appearance which are the spike glycoproteins on the viral envelope. SARS-CoV-2 belongs to the Coronavirus family, and *Betacoronavirus* subgenus. Members of this family often cause respiratory, enteric and neurologic diseases in mammals such as bats and even humans (Sharma et al., 2020a). The SARS-CoV-2 genome is about 29.9 kb, encoding four main structural proteins (spike (S), small envelope (E), membrane (M), and nucleocapsid (N) proteins), as well as other accessory proteins. The S protein is one of the main contributors to SARS-CoV-2 virulence, where it forms homotrimers, promoting the binding of the viral envelope to the ACE2 receptors on the host cell surface for entry (Zheng et al., 2020). ACE2 receptors are mainly expressed on type II alveolar epithelial cells, myocardial cells, kidneys, and are abundantly expressed on intestinal enterocytes. As a result, cell-free and macrophage-associated virus can travel from the lungs to other organs *via* the circulation, causing systemic infections (Hamming et al., 2004). Patients with critical SARS-CoV-2 infection exhibit multiple organ damage stemming from thromboembolic disease, hyper-coagulation, and the “cytokine storm” arising from overproduction of pro-inflammatory cytokines from infected monocytes and macrophages (Klok et al., 2020; Mondal et al., 2020; Tang et al., 2020).

Remdesivir is proposed to be a nucleoside analogue that inhibits viral RNA-dependent RNA polymerase (RdRp) to prevent viral RNA synthesis, thereby hampering virus replication in host cells. *In vitro* studies reported up to 6-log_10_ reduction of MHV with treatment using remdesivir at 11.1 μM (Agostini et al., 2018). A large double-blind, randomized, placebo-controlled trial of remdesivir in 1062 hospitalized adults with COVID-19 revealed shorter recovery time and lower mortality compared with the placebo group (Beigel et al., 2020). Another trial found that remdesivir did not provide significant antiviral effects in seriously ill patients (Wang et al., 2020).

Previous *in vitro* chloroquine treatment of Vero E6 cells infected with 2003-SARS-CoV demonstrated dose-dependent reduction of viral load by 40-80% (Vincent et al., 2005). Recent clinical trials of SARS-CoV-2-infected patients who received chloroquine phosphate treatment documented clinical improvements such as improved lung imaging, and shorter disease course (Gao et al., 2020). An open-label randomized trial found positive impact of chemoprophylaxis with oral hydroxychloroquine in reducing SARS-CoV-2 infection in young and healthy men (Seet et al., 2021). Chloroquine is proposed to affect the endocytic pathway by increasing pH levels within endosomes and lysosomes, thereby inhibiting proteases to prevent cleavage of the spike protein and subsequent virus entry into host cells (Satarker et al., 2020). SARS-CoV-2-infected patients at high risk of severe disease who were on doxycycline intervention showed improved clinical symptoms and enhanced recovery (Yates et al., 2020).

*In vitro* experiments observed a 99.8% reduction in viral RNA when SARS-CoV-2-infected Vero/hSLAM cells were treated with 5 μM ivermectin (Caly et al., 2020). One COVID-19 clinical trial reported that intervention with ivermectin achieved earlier virus clearance compared to placebo (9.7 versus 12.7 days), with decreased severity biomarkers (CRP and LDH) by day 7 (Ahmed et al., 2021). Another ivermectin trial involving non-severe COVID-19 patients documented reduction in anosmia/hyposmia, cough, viral loads, and IgG antibody titers (Chaccour et al., 2021). However, one double-blind randomized controlled trial found no significant difference in duration of symptoms (10 versus 12 days) in adults with mild COVID-19 treated with ivermectin versus placebo (Lopez-Medina et al., 2021). More recently, a randomized trial of hospitalized COVID-19 patients revealed that an oral high-dose ivermectin regimen was well-tolerated and led to antiviral activity that was dependent on plasma concentration of the drug (Krolewiecki et al., 2021). A recent meta-analysis of 15 trials indicated moderate-certainty evidence that large reductions in COVID-19 mortality are possible with ivermectin treatment (versus control) – early therapy may also decrease progression to severe disease (Bryant et al., 2021).

Combination therapy can target different pathways and processes during the coronavirus life-cycle to synergistically augment reduction in viral load. Hence, this strongly emphasizes the practical need to develop optimal combination therapies and increase their accessibility to COVID-19 patients worldwide.

This study explored the potential repurposing of four clinically approved drugs (remdesivir, chloroquine, ivermectin, doxycycline) to inhibit murine coronavirus and viral RNA replication, and to dampen excessive cytokine production. Given that it belongs to the same *Betacoronavirus* genus as SARS-CoV-2, murine hepatitis virus (MHV) was employed as a safe surrogate virus to infect the RAW264.7 mouse macrophage cell line (Pope et al., 1998) to mimic macrophage infection in COVID-19. The specific objectives were: (a) to compare the efficacy of monotherapy versus the efficacy of combined therapy with remdesivir to determine drug synergism; and (b) to investigate the effects of the drugs on cytokine production by infected macrophages.

## Materials and Methods

### Virus and Cell Cultures

All viral infection experiments were conducted using MHV strain A59 (MHV-A59; GenBank accession number AY910861) (Chiow et al., 2016). The RAW264.7 murine macrophage cell line was maintained at 37°C in RPMI-1640 medium supplemented with 10% fetal bovine serum (FBS). H2.35 murine liver cells were maintained at 35°C in Dulbecco’s modified Eagle medium (DMEM) with 10% FBS. Cells were seeded at a density of 200,000 cells per well on 24-well plates, and at a density of 15,000 cells per well on 96-well plates. Cells subjected to infection and/or drug treatment were monitored daily for any cytopathic effect (CPE).

### Drugs

Remdesivir (HY-104077; MedChemExpress, Monmouth Junction, NJ, USA) was prepared in 100 mM and 10 mM stock solutions in sterile water. Chloroquine (C6628; Sigma-Aldrich, St. Louis, MO, USA) and ivermectin (CAS 70288-86-7; Merck, Burlington, MA, USA) were prepared in 10 mM stock solutions in dimethyl sulfoxide (DMSO). Doxycycline (CAS 24390-14-5; Merck, Burlington, MA, USA) was prepared in 10 mM stock solution in sterile water. For treatment experiments, all drug dilutions were prepared in 0.5% DMSO with the respective cell culture medium.

### Cell Viability Assay

Cell viability was assessed using the CellTiter 96 AQueous One Solution Cell Proliferation (MTS) assay (Promega, Madison, WI, USA), according to the manufacturer’s instructions. RAW264.7 cells were incubated in the presence of increasing drug concentrations (0.16 μM, 0.8 μM, 4 μM, 20 μM, 100 μM) for 48 hours. Cell viability was determined using a microplate reader (Tecan, Mannedorf, Switzerland) with values normalized to those of untreated cells.

### Evaluation of Antiviral Inhibitory Activities of Monotherapy and Combination Therapy

Sub-confluent monolayers of RAW264.7 cells in 24-well plates were infected with MHV at multiplicity of infection (MOI) of 0.1 for 2 hours. The inoculum was then removed, and the cells were treated with the indicated concentrations of drugs for monotherapy or combination therapy for 48 hours. The cell supernatant was harvested at 48 hours post-infection, and subjected to virus plaque assay using H2.35 mouse liver cells to determine live coronavirus titers, and to real-time PCR to quantify the viral RNA loads. Each experiment was carried out in triplicates.

### Live Coronavirus Quantification by Plaque Assay

Live virus was quantified using plaque assay. Sub-confluent H2.35 cells were infected with the diluted supernatant samples for 1 hour. 1.2% Avicel BioPolymer (FMC, Philadelphia, PA, USA) in phosphate-buffered saline (PBS) was added to each well and incubated for 72 hours. The cells were fixed with 4% paraformaldehyde in PBS for 2 hours, and then stained with 1% crystal violet for 15 minutes.

### RNA Extraction, Viral RNA Quantification by Real-Time Reverse Transcription- Quantitative PCR

Cell supernatant was harvested from the experiments, and total viral RNA was purified using QIAamp Viral RNA Mini Kit (Qiagen, Hilden, Germany). RNA concentration was determined using the NanoDrop ND-1000 spectrophotometer. Reverse transcription was carried out at 35°C for 1 hour. Quantification of viral RNA was performed using MHV-NF forward and MHV-NR reverse primers for the specific detection of the N gene of MHV (5′-ACGCTTACATTATCWACTTC-3′ and 5′-GATCTAAATTAGAATTGGTC-3′, respectively). The qPCR was performed with FastStart Essential DNA Green Master using the LightCycler 96 instrument (Roche Diagnostics, Basel, Switzerland). To plot the standard curve, a range of positive controls with known plaque-forming units (PFU) of MHV was included. The thermal cycling conditions were as follows: 95°C for 600 s, followed by 55 cycles of 95°C for 10 sec, 40°C for 5 sec, 72°C for 8 sec, and a final 95°C for 10 sec, 65°C for 60 sec and 97°C for 1 sec.

### Determining Cytokine Protein Expression Profiles Using Luminex Multiplex Assay

To determine cytokine expression levels, multiplex assays using the Myokine 5-Plex Mouse ProcartaPlex Panel kit (Thermo Fisher Scientific, Waltham, MA, USA) were conducted according to the manufacturer’s instructions. This 5-plex kit quantified the following cytokines: IL-6, IL-10, IL-15, leukemia inhibitory factor (LIF), and TNF-α. Standard curves and values were measured on Luminex MAGPIX, and calculated using xPONENT 4.2 software for MAGPIX (with 30 beads being set as the detection limit).

### Statistical Analyses

Non-linear regression and curve-fitting parameter analyses were performed to calculate inhibitory concentration values using GraphPad Prism 7 (GraphPad Software, San Diego, CA, USA). Statistical significance and *P*-values were calculated by one-way ANOVA using Dunnett’s multiple comparison test. Each error bar of dose-response curves represents the standard error of mean (SEM) of three technical replicates.

## Results

### Monotherapy of Drugs Inhibits MHV Infection of RAW 264.7 Macrophages With Minimal Toxicity

**Figure 1:**
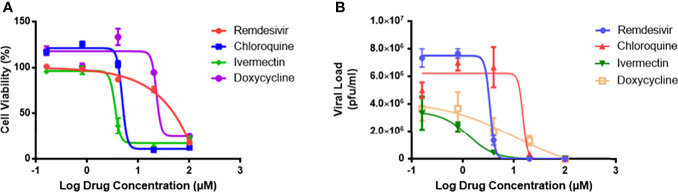
The drug cytotoxicity profiles of RAW264.7 macrophages, and dose-response curves of MHV-infected macrophages. **(A)** Cytotoxicity profiles of the four individual drugs on RAW264.7 cells as measured by MTS assay after 48 hours exposure. Percentage of cell viability was normalized to untreated cells and blank control. Experiments were performed in quadruplicates. **(B)** Viral inhibitory activities of the four drugs against MHV infection of RAW264.7 macrophages. Live coronavirus titers (pfu/ml) were quantified by plaque assays performed in triplicates. The dose-response curves were fitted using the non-linear regression method, and IC50 values were calculated using the Prism 7 software. Error bars represent standard error of the mean (SEM).

To evaluate the safety profile of the drugs, cytotoxicity assays were carried out (**Figure 1A**), and the results expressed as cell viability curves. To evaluate the effectiveness of monotherapy of the individual drugs, increasing concentrations of the drugs were tested against MHV infection (**Figure 1B**). **Table 1** summarizes the 50% cytotoxic concentration (CC50), 50% inhibitory concentration (IC50), and selectivity index (SI) from the monotherapy experiments. In treatment of RAW264.7 cells, ivermectin exhibited the highest SI index, indicating its high safety and efficacy ratio as a treatment option. Based on these determined IC50 and CC50 ranges (**Table 1**), downstream monotherapy and combination therapy experiments were subsequently performed using the following drug concentrations above their respective IC50 values with high cell viability and low cytotoxicity: remdesivir at 6 µM, chloroquine at 15 µM, ivermectin at 2 µM, and doxycycline at 15 µM.

**Table 1:** Mean values of CC50, IC50 and selectivity index (SI = CC50/EC50) of remdesivir, chloroquine, ivermectin and doxycycline treatment of RAW264.7 macrophages.

	Remdesivir	Chloroquine	Ivermectin	Doxycycline
CC50	58.12	48.79	37.38	72.07
IC50	3.38	14.92	1.26	9.60
Selectivity index (SI)	17.18	3.27	29.64	7.50

To further verify the drug cytotoxicity and antiviral efficacy in another cell line, we selected and assessed remdesivir and chloroquine treatment in H2.35 murine liver cells without and with MHV infection. As shown in **Supplementary Figure 1**, remdesivir also exhibited a significantly higher selectivity index (204.2) versus that of chloroquine (10.6) in mouse liver cells.

### Combination Therapy of Remdesivir With Ivermectin or With Doxycycline Is Synergistic in Reducing Coronavirus Load and Restoring Healthy Cell Morphology

**Figure 2:**
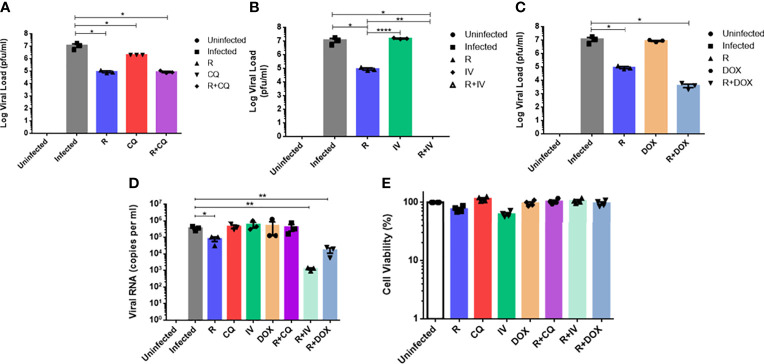
Comparison of antiviral activities of monotherapy versus combination therapy using remdesivir with chloroquine, ivermectin and doxycycline against MHV infection of RAW264.7 macrophages. Remdesivir (R) was tested at a concentration of 6 µM alone or in combination with the other three drugs. Uninfected cells, and infected but untreated cells served as controls. Live coronavirus titers were quantified using plaque assays. **(A)** Infected cells were treated with 15 µM of chloroquine (CQ) alone or combined with R. **(B)** Infected cells were treated with 2 µM of ivermectin (IV) alone or combined with R. **(C)** Infected cells were treated with 15 µM of doxycycline (DOX) alone or combined with R. **(D)** Quantification of MHV RNA loads (copies per ml) in extracts of supernatants from the above experiments using real-time RT-qPCR. Error bars represent SEM. Statistical significance was determined by one-way ANOVA with Dunnett’s multiple comparison, and *P*-values denoted by asterisks: *P < 0.05; **P < 0.01; ****P < 0.0001. **(E)** Cell viability profiles of uninfected and drug-treated cells at tested concentrations. Mean percentages of cell viability were normalized to untreated cells and blank controls. Experiments were carried out in quadruplicates.

To determine the efficacies of the drug combinations (together with remdesivir), monotherapy experiments at the same concentrations were compared against those of combination treatments. As shown in **Figures 2A–C**, monotherapy at 6 µM remdesivir yielded a significant 2-log_10_ reduction in live virus (*P* < 0.05). Chloroquine monotherapy demonstrated a significant 1-log_10_ viral reduction (*P* < 0.05), whereas monotherapy with ivermectin or doxycycline showed no statistically significant difference from untreated infected cells (*P* > 0.05).

There are various primary methods for antimicrobial synergy testing, including the multiple-combination antimicrobial test or MCAT (Doern, 2014). MCAT is an accepted and recognized method for simultaneously assessing combinations of two or more drugs at fixed concentrations against a test organism at a specified inoculum (Aaron et al., 2000). This technique can demonstrate whether the combined effect of two drugs *in vitro* can exhibit synergism (i.e. an effect larger than the sum of the effect of each independent drug); or additive effect (i.e. an effect equal to the sum of the individual drug effects); or antagonism (i.e. an effect smaller than the sum of the independent drug effects) (Greco et al., 1995).

Combination therapy with remdesivir and chloroquine (**Figure 2A**) exhibited a significant 2-log_10_ reduction in live viral load (*P* < 0.05), which was similar to remdesivir monotherapy, indicating that the antiviral effect of this combination was largely due to remdesivir, without drug synergism. In contrast, combination of remdesivir with ivermectin (**Figure 2B**) was the most effective and completely inhibited viral replication, with a significantly potent reduction of 7-log_10_ of live virus, i.e. an additional reduction of 5-log_10_ of live virus compared to remdesivir monotherapy (*P* < 0.01), thus revealing evidence of drug synergism. Some synergism was also observed in the combination of remdesivir with doxycycline (**Figure 2C**), where a further reduction of 1-log_10_ of live virus titer was achieved compared to remdesivir alone.

Real-time RT-qPCR quantification of MHV RNA displayed a similar trend to the virus plaque assays (**Figure 2D**). Remdesivir monotherapy attained a significant reduction of about 1-log_10_ of viral RNA (*P* < 0.05), whereas monotherapy with the other drugs did not lead to significant reduction in viral RNA. Combination of remdesivir with ivermectin or with doxycycline culminated in the most significant reductions in viral RNA loads of 2.5-log_10_ and 1.5-log_10_ respectively (*P* < 0.01), thus reiterating drug synergism. These results are congruent with the live virus titers which highlighted that remdesivir in combination with ivermectin or doxycycline could significantly decrease both live virus and viral RNA loads. Therefore, the most effective drug combination that exhibited highly potent synergism was remdesivir with ivermectin, followed by remdesivir with doxycycline.

**Figure 2E** shows high cell viability of RAW264.7 macrophages for monotherapy and combination therapy with the drugs at the tested concentrations. It is notable that the mean cell viability was above 95% for each of the drug combinations evaluated.

As shown in the **Supplementary Figure 2**, the four monotherapy treatments (**Supplementary Figures 2C–F**) exhibited cell morphologies that were more similar to the infected untreated control cells (**Supplementary Figure 2B**) with prominent CPE. In contrast, the combination therapies (**Supplementary Figures 2G–J**) displayed cell morphologies that resembled the uninfected control (**Supplementary Figure 2A**), with rounder and smoother cell surface layers. This suggests that the combination therapy (particularly remdesivir and ivermectin) was able to restore cell appearance to a healthy state, in contrast to monotherapy which showed extensive CPE.

### Combination Therapy With Remdesivir and Ivermectin Decreases the Levels of Key Cytokines Associated With the Cytokine Storm

**Figure 3:**
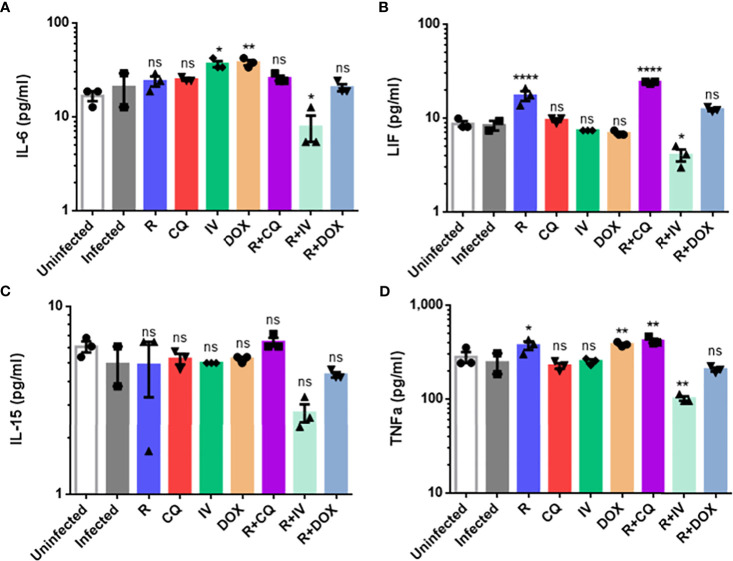
Cytokine protein expression profiles of monotherapy versus combination therapy against MHV infection of RAW264.7 macrophages. Remdesivir (R), chloroquine (CQ), ivermectin (IV) and doxycycline (DOX) treatments were evaluated. Cytokine protein levels (pg/ml) of **(A)** IL-6, **(B)** leukemia inhibitory factor or LIF, **(C)** IL-15, **(D)** TNF-α were quantified using the Myokine 5-Plex Mouse ProcartaPlex Panel in drug-treated infected samples compared to controls (uninfected cells, and infected but untreated cells). IL-10 cytokine levels were below the detection limit, and were thus omitted. The data represent means and SEM from triplicate experiments. Statistical significance was determined by one-way ANOVA with Dunnett’s multiple comparison against the infected untreated control, and denoted by asterisks: *P < 0.05; **P < 0.01; ****P < 0.0001. ns, not statistically significant.

Given that certain drugs (individually or in combination) could inhibit live virus and viral RNA, we next analyzed the drug effects on cytokine levels in infected macrophages. The protein expression levels of five key cytokines were quantified, namely IL-6, IL-10, IL-15, LIF and TNF-α (**Figure 3**). The IL-10 readings were below the detection limit and out of range – thus, the IL-10 results were omitted.

As shown in **Figure 3**, combination treatment with remdesivir and ivermectin was found to significantly decrease IL-6 (*P* < 0.05), LIF (*P* < 0.05), and TNF-α (*P* < 0.01). This drug combination also resulted in lower IL-15 level, although it was not statistically significant. To verify these findings, a biological replicate experiment yielded a reproducible trend in the decreased cytokines (data not shown). This suggests that the additional 5-log_10_ reduction in live virus titer (**Figure 2B**) was complemented by amelioration of cytokine production to exert anti-inflammatory effects. It is noteworthy that IL-6 and TNF-α are significantly elevated in patients with severe COVID-19, and that LIF is an IL-6 cytokine that can activate the JAK/STAT and MAP kinase pathways (Traber et al., 2017).

## Discussion

Bronchoalveolar lavage fluid from severe COVID-19 patients contain abundant mononuclear phagocytes and inflammatory monocyte-derived macrophages. Autopsy analyses of COVID-19 patients reveal the presence of SARS-CoV-2 viral particles and antigen within lymph node and splenic macrophages. Aberrant mononuclear phagocyte activation or macrophage activation syndrome may lead to the cytokine release syndrome or “cytokine storm” which culminates in excessive inflammation and coagulopathy associated with COVID-19 severity and mortality (Merad and Martin, 2020; Sharma et al., 2021b). SARS-CoV-2-infected macrophages thus contribute to viral spread, exaggerated inflammation, and activation-induced lymphocytic cell death (Park, 2020).

In RAW264.7 macrophages, SARS-CoV-2 spike S1 induces pro-inflammatory responses (TNF-α expression) which can be markedly suppressed by RNA interference of TLR4 (Shirato and Kizaki, 2021). The related SARS-CoV spike protein can induce TNF-α and IL-6 expression by activating the NF-κB signaling pathway in RAW264.7 cells (Wang et al., 2007).

Besides MHV, the RAW264.7 line also supports replication of other viral pathogens, including Japanese encephalitis virus, mengovirus, and pneumonia virus of mice (PVM) (Murali-Krishna et al., 1995; Martin et al., 2000; Dyer et al., 2007). On the other hand, the murine lung alveolar macrophage MH-S cell line does not support replication of PVM (Brenner et al., 2016). In one study, SARS‐CoV‐2 challenge of human alveolar macrophages (AMs) does not result in the release of free SARS‐CoV‐2 RNAs and proteins into the cytoplasm, suggesting that AMs are not productively infected (Dalskov et al., 2020).

M1 phenotype (pro-inflammatory) macrophages arise as a result of classical activation *via* stimulation of toll-like receptors through ligands, including microbial stimuli (e.g. lipopolysaccharide or LPS) and certain cytokines (such as IFN-γ and TNF-α). M1 macrophages produce inflammatory mediators (e.g. IL-6, TNF-α, nitric oxide), and significantly contribute to the immune response (Sica et al., 2015; Smith et al., 2016). For example, one regulatory mechanism of M1 polarization and activation of RAW264.7 macrophages is triggered by activating the TLR4 and NF-κB signaling pathway (Shi et al., 2020). Interestingly, in contrast to M2 AMs, M1 AMs have a lower endosomal pH that favors membrane fusion, permits entry of viral RNA from the endosome into the cytoplasm where SARS-CoV-2 replicates – the virus is then packaged and released to facilitate infection of the lungs (Lv et al., 2021).

RAW264.7 and J774.1 macrophage cell lines are well-established model systems in cell biology and immunology research. Bacterial LPS induces dose-dependent secretion of pro-inflammatory cytokines (IL-6, TNF-α, MCP-1) in both RAW264.7 and J774.1 cells (Nativel et al., 2017). Moreover, in both macrophage cell lines infected with herpes simplex virus 1 and treated with inhibitory CpG, similar viral induction of cytokine expression involving TLR9 is observed (Malmgaard et al., 2004). Sun et al. (2020) also showed that estradiol can promote trained immunity in both RAW264.7 and J774.1 by using TNF-α and IL-1β as the marker of inflammation. Considering the above reasons, we therefore focused on RAW264.7 as our working macrophage cell line model in our experiments.

Efficacious broad-spectrum pan-coronavirus antivirals are needed to treat the highly infectious and virulent SARS-CoV-2, as well as emerging zoonotic coronaviruses to prepare for future epidemics and even pandemics. Although there are many studies and clinical trials testing the efficacy of the four tested drugs, very few explore the combination with remdesivir, which is the most studied antiviral for SARS-CoV-2 treatment.

In this study, remdesivir alone at 6 µM was able to achieve a 2-log_10_ live viral reduction, but was exceptionally synergistic when combined with ivermectin at 2 µM, culminating in an extremely potent 7-log_10_ reduction in viral load, with no visible virus plaques even at the highest virus concentration tested. A similar trend was corroborated by the synergistic reduction of 2.5-log_10_ of viral RNA with the combination of remdesivir and ivermectin, compared to the corresponding monotherapy. Hence, the additional 5-log_10_ of live virus reduction arising from synergism may be attributed to mechanisms other than inhibition of viral RNA production. It is notable that in hospitalized COVID-19 patients, infectious virus was readily isolated from samples of the throat or lung during the first week of symptoms. However, when symptoms generally subsided after day 8, live virus could not be cultured despite ongoing high viral RNA loads detectable in throat swabs well into the second week (Wölfel et al., 2020). Another study supported the *in vitro* synergistic interaction between remdesivir and ivermectin resulting in enhanced antiviral activity against SARS-CoV-2 (Jeffreys et al., 2020).

Strikingly, the remdesivir and ivermectin combination culminated in the most significant and consistent reduction of IL-6, LIF and TNF-α, thus highlighting its important impact on inhibiting the production of pro-inflammatory cytokines from infected macrophages. This notable anti-inflammatory characteristic of this drug combination is relevant in view of the need to ameliorate the “cytokine storm” especially in patients with severe coronavirus infections, to improve associated complications, and to delay disease progression (Narasaraju et al., 2020). In addition, the drug combinations also helped to improve morphology of the infected cells (**Supplementary Figure 2**) compared to the monotherapy and infected controls, which may promote and restore proper cell growth following coronavirus infection. While SARS-CoV-2 can infect human macrophages, it is well-appreciated that these cells are not a primary target of SARS-CoV-2 infection. One mechanism to explain the “cytokine storm” associated with COVID-19 is that SARS-CoV-2 pathogen-associated molecular patterns trigger IL-6 production followed by hyper-activation of the NF-κB pathway in both immune and non-immune cells, culminating in the establishment of the IL-6 amplifier inflammatory circuit (Hojyo et al., 2020).

The mode of action of remdesivir in coronavirus infection is primarily targeting RdRp-mediated RNA synthesis, thus preventing virus replication and diminishing viral load (Agostini et al., 2018). Notably, remdesivir also possesses broad-spectrum antiviral inhibition of human and zoonotic coronaviruses with divergent RdRp (Brown et al., 2019). A proposed antiviral mechanism of ivermectin is the inhibition of nuclear translocation of viral proteins which is mediated by host importin α/β1 heterodimerization (Wagstaff et al., 2012). The preformed heterodimer can also be destabilized by ivermectin. In addition, ivermectin can hinder heterodimer formation *via* binding to the importin-α armadillo repeat domain to affect thermal stability and α-helicity of importin-α, thereby preventing its binding to importin β1 (Yang et al., 2020). One possible mode of action of ivermectin is through blocking the nuclear localization signal of importin-α (Kinobe and Owens, 2021). Viral protein entry into the nucleus is thus impeded, which impairs viral inhibition of the host anti-viral response, culminating in more effective viral clearance (Caly et al., 2020). Computational modelling of doxycycline and SARS-CoV-2 proteins identified this drug to be a potential inhibitor of the 3C-like main protease that mediates the maturation of non-structural proteins, potentially restricting viral replication (Wu et al., 2020). In mice with influenza virus pneumonia, doxycycline treatment ameliorates acute lung injury with significantly diminished inflammation, protein leakage, and matrix metalloproteinase activity in the lungs (Ng et al., 2012).

Going forward, more studies and trials are warranted to better understand the underlying mechanisms by which ivermectin and doxycycline synergize with remdesivir to exert antiviral activities against coronaviruses in multiple cell lines. Importantly, by targeting different viral and/or host processes, effective drug combinations can lower the therapeutic concentrations of drugs compared to monotherapy, thus reducing treatment toxicity and costs. Moreover, such drug combinations can minimize drug-resistant virus strains which have higher propensity to emerge with monotherapy (Sun et al., 2016). Indeed, combination treatment with remdesivir plus baricitinib (a Janus kinase inhibitor) was superior to remdesivir alone in improving clinical status and recovery among COVID-19 patients (Kalil et al., 2021). A multi-center study reported that four approved drugs against hepatitis C that inhibit the papain-like protease of SARS-CoV-2 can enhance the antiviral effects of remdesivir by ten-fold *in vitro* (Bafna et al., 2021). Synergy between remdesivir and emetine has also been documented (Choy et al., 2020). Given that remdesivir is relatively more expensive, its potential combination at lower doses together with more affordable drugs can lower the overall treatment cost, thus offering cheaper but effective options especially to lower- and middle-income countries which are already suffering from existing epidemics such as tuberculosis (Bong et al., 2020).

## Conclusion

In summary, this *in vitro* study has provided evidence that the combination of remdesivir and ivermectin was highly synergistic and potent, culminating in striking reduction in live murine coronavirus replication and viral RNA synthesis. Furthermore, the efficacy and beneficial effects of this combination therapy also included immunomodulatory effects *via* inhibition of cytokine production, and improving cell morphology of infected macrophages.

The limitation of our study is that antiviral activities of drugs were investigated only against MHV in the RAW264.7 macrophage cell culture model. For direct comparison versus MHV, future detailed studies are thus warranted to evaluate the potential utility of this drug combination against SARS-CoV-2 and other coronaviruses (e.g. SARS-CoV, MERS-CoV, HCoV-229E, HCoV-OC43), such as multi-dose checkerboard experiments to ascertain additive effects of drugs (Bakowski et al., 2021). This caveat is also critically important given the observations of cell line-dependent compound efficacy against SARS-CoV-2 infection reported in many previous SARS-CoV-2 antiviral studies. For example, Vero cells, human Huh-7.5 liver cancer cells and human Calu-3 lung epithelial cells exhibit major differences in sensitivity to drugs with antiviral activity due to the distinct entry pathways utilized by SARS-CoV-2 in these cell lines (Dittmar et al., 2021). Also necessary are *in vivo* experiments using suitable animal model(s) of COVID-19 before progressing onto clinical trials in SARS-CoV-2 infected patients.

## Data Availability Statement

The raw data supporting the conclusions of this article will be made available by the authors, without undue reservation.

## Author Contributions

VC and YT conceptualized and designed the research project, and analyzed the data. All experiments were carried out by YT. All authors contributed to the article and approved the submitted version.

## Funding

This study was supported by a research grant of the National University of Singapore.

## Conflict of Interest

The authors declare that the research was conducted in the absence of any commercial or financial relationships that could be construed as a potential conflict of interest.

## Publisher’s Note

All claims expressed in this article are solely those of the authors and do not necessarily represent those of their affiliated organizations, or those of the publisher, the editors and the reviewers. Any product that may be evaluated in this article, or claim that may be made by its manufacturer, is not guaranteed or endorsed by the publisher.

## Supplementary Material

Supplementary Figure 1 The drug cytotoxicity profiles of H2.35 murine liver cells, and dose-response curves of MHV-infected H2.35 cells. **(A)** Cytotoxicity profiles of remdesivir and chloroquine on H2.35 cells as measured by MTS assay after 48 hours exposure. Percentage of cell viability was normalized to untreated cells and blank control. Experiments were performed in quadruplicates. **(B)** Viral inhibitory activities of remdesivir and chloroquine against MHV infection of H2.35 cells. Live coronavirus titers (pfu/ml) were quantified by plaque assays performed in triplicates. The dose-response curves were fitted using the non-linear regression method, and IC50 values were calculated using the Prism 7 software. Error bars represent standard error of the mean (SEM). **(C)** Mean values of CC50, IC50 and selectivity index (SI = CC50/EC50) of remdesivir and chloroquine treatment of H2.35 cells.

Supplementary Figure 2 Representative morphological features of RAW264.7 macrophages under different infection and/or treatment conditions. Microscopic images were captured by the EVOS XL microscope at 10× magnification. The scale bar represents 200 μm. **(A)** Uninfected control cells. **(B)** MHV-infected control cells. Monotherapy of MHV-infected macrophages using: **(C)** remdesivir alone, **(D)** chloroquine alone, **(E)** ivermectin alone, **(F)** doxycycline alone. Combination therapy of MHV-infected macrophages using: **(G)** remdesivir and chloroquine, **(H)** remdesivir and ivermectin, **(J)** remdesivir and doxycycline.
