# Supplementary figures and images for: Combination Treatment With Remdesivir and Ivermectin Exerts Highly Synergistic and Potent Antiviral Activity Against Murine Coronavirus Infection
Source: Front Cell Infect Microbiol. 2021 Jul 30;11:700502. doi: 10.3389/fcimb.2021.700502 (PMC8362885; doi:10.3389/fcimb.2021.700502)

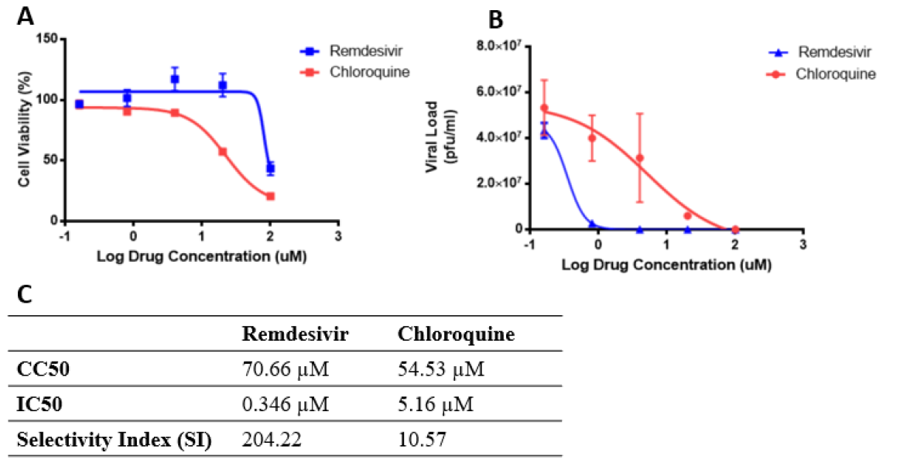

Supplement: Supplementary Figure 1 — The drug cytotoxicity profiles of H2.35 murine liver cells, and dose-response curves of MHV-infected H2.35 cells. (A) Cytotoxicity profiles of remdesivir and chloroquine on H2.35 cells as measured by MTS assay after 48 hours exposure. Percentage of cell viability was normalized to untreated cells and blank control. Experiments were performed in quadruplicates. (B) Viral inhibitory activities of remdesivir and chloroquine against MHV infection of H2.35 cells. Live coronavirus titers (pfu/ml) were quantified by plaque assays performed in triplicates. The dose-response curves were fitted using the non-linear regression method, and IC50 values were calculated using the Prism 7 software. Error bars represent standard error of the mean (SEM). (C) Mean values of CC50, IC50 and selectivity index (SI = CC50/EC50) of remdesivir and chloroquine treatment of H2.35 cells. [file Image_1.png]

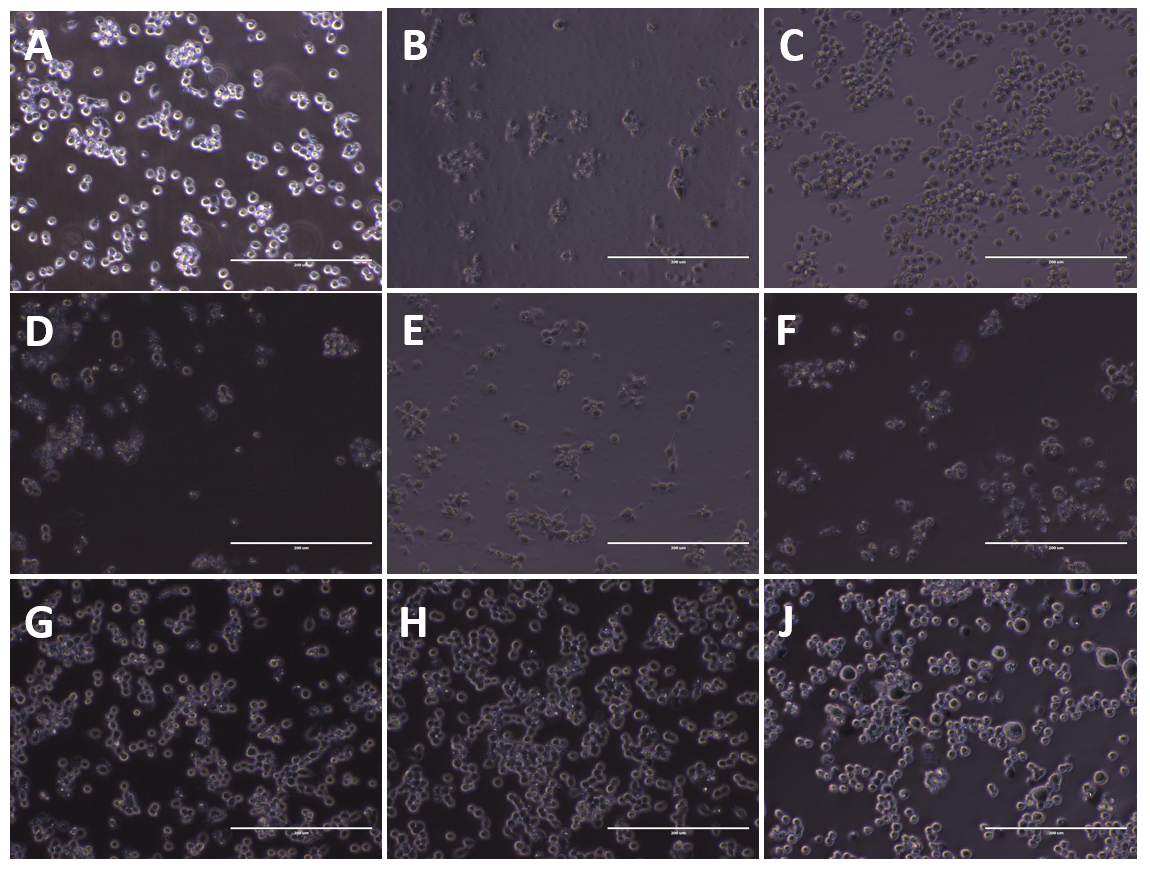

Supplement: Supplementary Figure 2 — Representative morphological features of RAW264.7 macrophages under different infection and/or treatment conditions. Microscopic images were captured by the EVOS XL microscope at 10× magnification. The scale bar represents 200 μm. (A) Uninfected control cells. (B) MHV-infected control cells. Monotherapy of MHV-infected macrophages using: (C) remdesivir alone, (D) chloroquine alone, (E) ivermectin alone, (F) doxycycline alone. Combination therapy of MHV-infected macrophages using: (G) remdesivir and chloroquine, (H) remdesivir and ivermectin, (J) remdesivir and doxycycline. [file Image_2.tif]
